# Transcriptome Analysis of *Catharanthus roseus* for Gene Discovery and Expression Profiling

**DOI:** 10.1371/journal.pone.0103583

**Published:** 2014-07-29

**Authors:** Mohit Verma, Rajesh Ghangal, Raghvendra Sharma, Alok K. Sinha, Mukesh Jain

**Affiliations:** Functional and Applied Genomics Laboratory, National Institute of Plant Genome Research, Aruna Asaf Ali Marg, New Delhi, India; National Key Laboratory of Crop Genetic Improvement, China

## Abstract

The medicinal plant, *Catharanthus roseus*, accumulates wide range of terpenoid indole alkaloids, which are well documented therapeutic agents. In this study, deep transcriptome sequencing of *C. roseus* was carried out to identify the pathways and enzymes (genes) involved in biosynthesis of these compounds. About 343 million reads were generated from different tissues (leaf, flower and root) of *C. roseus* using Illumina platform. Optimization of *de novo* assembly involving a two-step process resulted in a total of 59,220 unique transcripts with an average length of 1284 bp. Comprehensive functional annotation and gene ontology (GO) analysis revealed the representation of many genes involved in different biological processes and molecular functions. In total, 65% of *C. roseus* transcripts showed homology with sequences available in various public repositories, while remaining 35% unigenes may be considered as *C. roseus* specific. *In silico* analysis revealed presence of 11,620 genic simple sequence repeats (excluding mono-nucleotide repeats) and 1820 transcription factor encoding genes in *C. roseus* transcriptome. Expression analysis showed roots and leaves to be actively participating in bisindole alkaloid production with clear indication that enzymes involved in pathway of vindoline and vinblastine biosynthesis are restricted to aerial tissues. Such large-scale transcriptome study provides a rich source for understanding plant-specialized metabolism, and is expected to promote research towards production of plant-derived pharmaceuticals.

## Introduction

*Catharanthus roseus*, popularly known as Madagascar periwinkle, is a medicinal plant which belongs to family Apocyanaceae. The plant is diploid (2n = 16) and native to islands of Madagascar, but now grown in many tropical countries as ornamental plant [1]. *C. roseus* is well known for its pharmacological importance as it produces more than 130 terpenoid indole alkaloids (TIAs) including vinblastine and vincristine, which are widely used in anti-cancer chemotherapies [2], [3]. Most tissues of *C. roseus* are known to produce alkaloids and no other single plant is known to produce such a wide spectrum of alkaloids [4]. The plant is known to treat diabetes also, due to hypoglycemic properties in its tissue extracts [5]. Moreover, roots of *C. roseus* are known to accumulate ajmalicine and serpentine which help controlling blood pressure and cardio-vascular disorders [6].

Alkaloid biosynthetic pathways are highly branched and complex, with wide differences in alkaloid composition between underground and aerial tissues. TIAs have high commercial value because they are produced by plants in very low amounts and its infusion is very difficult. The common precursor of TIAs, strictosidine, is the central intermediate formed by the condensation of tryptamine (product of shikimate pathway) and secologanin (product of non-mevalonate pathway) involving strictosidine synthase (STR). Pharmacologically important alkaloids, vinblastine and vincristine (found only in aerial tissues) are synthesized *in vivo* by the condensation of vindoline and catharanthine, both of which are obtained from branch-point intermediate cathenamine. Biochemical pathway resulting in formation of vindoline is specifically present in well differentiated aerial tissues of the plant, but not in roots and cell cultures, thereby marking the presence of tissue-specific TIA pathway in *C. roseus* [7]–[11].

In recent years, next generation sequencing has become the method of choice for fast and cost-effective transcriptome characterization for non-model plants [12]–[15]. Earlier, a large-scale transcriptomic resource from three medicinal plants (*Camptotheca acuminate*, *Catharanthus roseus* and *Rauvolfia serpentina*) have been developed for elucidating monoterpene indole alkaloid (MIA) pathways [16]. Recently, Van Moerkercke et al. [17] used RNA-seq approach to construct metabolic pathway database, CathaCyc, for *C. roseus*. Such databases can facilitate identification of key regulator(s) for metabolic pathway engineering. To add on to existing resources, in this study, we generated *C. roseus* transcriptome by assembling RNA-seq data generated from *C. roseus* tissues (leaf, flower and root) and merged it with previously reported *C. roseus* transcripts. The updated comprehensive *C. roseus* transcriptome was screened for simple sequence repeats (SSRs) which might be helpful in development of functional molecular markers. We also identified transcription factor (TF) encoding transcripts in *C. roseus* transcriptome as only few TFs are known, which regulate TIA pathway genes. Expression analysis of genes involved in TIA pathways was also undertaken to reveal their tissue-specific expression. Gene ontology (GO) enrichment analysis highlighted the tissue-preferential/specific expression of transcripts in various biological processes. These data will provide a framework for further functional analysis of genes involved in biosynthesis of important alkaloids.

## Results

### Transcriptome sequencing and preprocessing of data

To generate the transcriptome of *C. roseus*, three tissue samples (leaf, root and flower) were subjected to next generation sequencing using Illumina platform. Of the total ∼347 million reads generated from all the tissue samples, about 343 million reads were found to be of high quality after filtering with NGS QC Toolkit (Table S1), having average Phred quality score of at least 30 at each base position. As the short reads obtained may be redundant (due to PCR amplification at library preparation step) and their assembly needs a high-end server with high random access memory (RAM). Therefore, duplicate reads from each sample were removed and about 230 million non-redundant (NR) reads were obtained (Table S1).

### Optimization and validation of transcriptome assembly

To generate an optimal transcriptome assembly of *C. roseus*, we systematically compared the performance of various *de novo* short read assembly tools, including Velvet, Oases and ABySS. *De novo* assembly of total (343,384,084) and NR (230,715,698) high quality reads was performed employing a two-step approach. In the first step, primary assembly (best k-mer assembly) was generated using Velvet, Oases and ABySS at different k-mer lengths ranging from 31 to 95 (Table S2 and S3). On the basis of several parameters described earlier [18], assemblies obtained from respective assemblers at different k-mers were compared. Assemblies generated by Velvet showed a gradual increase in N50 and average read length with the k-mer length, best being at k-93 (Table S2A and S3A). Similarly, assembly at k-93 (from total reads; Table S2C) and k-87 (from NR reads; Table S3C) had higher N50 and average read lengths, and were considered to be the best assembly generated from ABySS. On the other hand, choosing the best assembly generated from Oases (using NR dataset) was a tricky task, as there was not much difference in N50 and average lengths at different k-mers (Table S2B). Finally, assembly at k-57 was selected, which had an optimal assembly size (number of contigs; Table S3B) and minimum redundant unigenes. Whereas, assembly of Oases at k-61 generated from total dataset had higher N50 and average lengths (Table S2B). By taking all the assembly parameters into consideration along with BLAST results, assembly generated by Oases at k-57 using NR short read dataset (NR-Oases k-57), was considered to be the best, which generated 42909 contigs (≥250 bp) of 1161 bp average length and 1990 bp N50 length (Table 1).

**Table 1:** Assembly optimization/validation of Illumina data of *C. roseus*.

	Total high quality reads (best k-mer)				Non-redundant high quality reads (best k-mer)				MPGR assembly	Merged assembly^2^
	ABySS(k_87)	Velvet(k_93)	Oases(k_61)	Oases(k_61)	ABySS(k_87)	Velvet(k_93)	Oases(k_57)	Oases(k_57)		
**Number of contigs**	133650	39010	128716	106736	71190	39276	53017	42909	86726	59220
**Total size (Mb)**	72.78	25.38	165.01	160.92	57.62	25.62	51.86	49.81	107.79	76.03
**Minimum length (bp)**	100	185	100	250	100	185	107	250	251	250
**Maximum length (bp)**	15524	10893	17071	17071	15524	10913	17112	17112	12048	17141
**Average length (bp)**	544.6	650.7	1282	1507.8	809.5	652.3	978.2	1160.8	1243	1283.9
**N50 length (bp)**	1087	848	2161	2205	1400	841	1911	1990	1683	2115
**Contigs with significant similarity** 1	63706	28142	76866	73292	41643	28477	22593	21275	66788	32666

1 Similarity search was done against TAIR10 proteome.

2 Assembly generated by merging best k-mer with MPGR transcriptome using TGICL.

Gongora-Castillo et al. [16] followed a robust approach to generate a comprehensive *C. roseus* transcriptome from different tissues and treatments. Our second step involved merging of primary assembly (best k-mer; NR-Oases k-57) with previously existing *C. roseus* assembly (MPGR de novo assembly; [16]). Our earlier studies have shown that TGICL software generates optimal merged assemblies [15], [19]. Unigenes from both the assemblies were size selected (≥250 bp) and transcript isoforms from MPGR assembly were removed (retaining the longest isoform) before subjecting to assembly using TGICL program. The merged assembly resulted in a total of 59220 contigs with improved average length (1284 bp) and increased N50 read length (2115 bp; Table 1). Overall, the merged assembly was found to be much better than those reported previously [16], [20].

To assess the quality of *C. roseus* transcriptome thus obtained, we checked for the presence of publicly available *C. roseus* sequences in recently assembled transcriptome. Out of 287 full-length protein sequences (downloaded from NCBI), 220 (77%) were found to be present in the assembled transcriptome. Moreover, we also checked for the genes involved in various biochemical pathways and found that all the 108 genes previously reported by Van Moerkercke et al. [17] were represented in our transcriptome data. Similarly, all the 30 enzymes (genes) known to be involved in TIA bio-synthesis [17], were also present in the *C. roseus* transcriptome generated in this study.

As compared with earlier reported transcriptome assemblies of *C. roseus*, we obtained nearly 19% (MPGR assembly) [16] and 42% (CathaCyc) [17] novel transcripts in our assembly. *C. roseus* belongs to the clade Asterids, and genome of three plant *species* of this clade, including *Solanum tuberosum* (potato), *Solanum lycopersicum* (tomato) and *Sesamum indicum* (sesame) have been sequenced so far. A BLAST analysis of *C. roseus* transcriptome against proteomes of tomato, potato, cucumber, grapevine and Arabidopsis, and transcriptomes of six known alkaloid producing plants (*Atropa belladonna*, *C. acuminata*, *Cannabis sativa*, *R. serpentine*, *Rosmarinus officinalis* and *Valeriana officinalis*) revealed higher similarity of *C. roseus* transcripts with *R. officinalis* (60.9%) followed by tomato (58.7%), potato (56.3%), cucumber (56.2%) and grapevine (56.1%) (Fig. S1). Further, reciprocal BLAST analysis with annotated protein sequences from closest reference genomes (tomato, potato, cucumber and grapevine) and Arabidopsis showed that, although Arabidopsis happens to be distantly related to *C. roseus* in phylogenetic tree but had the highest number of orthologs (15252) as compared to tomato (12118) and potato (11263), which belong to the same clade (Asterids) as that of *C. roseus* (Fig. S2). This may be due to availability of better genome annotation of the model plant Arabidopsis. Cucumber and grapevine had the least number of orthologs in *C. roseus*, because they belong to different clades.

*C. roseus* transcripts generated above were designated as *C. roseus* tentative consensus (Cr_TC) and were assigned a unique identifier number from Cr_TC00001 to Cr_TC59220. The whole transcriptome sequence is available at Catharanthus Transcriptome Sequence web page (http://nipgr.res.in/mjain.html?page=catharanthus). The total size of transcriptome is ∼76 Mb with nearly 65% of the transcripts longer than 500 bp and more than 40% transcripts larger than 1000 bp (Fig. S3). Average GC content of *C. roseus* transcriptome was little lower (40.65%) than Arabidopsis (42.5%; Fig. S4), and comparable with that of soybean (40.9%) and chickpea (40.3%) [18]. Whereas, average GC content of rice was much higher (55%) with respect to *C. roseus* and other dicot plant species analyzed.

### Functional annotation of *C. roseus* transcriptome

For comprehensive annotation of *C. roseus* transcripts, similarity search was performed against several public databases sequentially. We were able to annotate 38380 (65%) unigenes with confidence (e-value≤1E-05), while others were considered to be *C. roseus* - specific which may be involved in various important biochemical pathways, whose intermediates and enzymes involved have not been catalogued in public repositories as of now. The putative function assigned to the transcripts is available at Catharanthus Transcriptome Sequence web page. Based on their similarity with Arabidopsis genes, *C. roseus* transcripts were assigned GOSlim terms under biological process, molecular function and cellular components categories (Fig. S5A). Among the biological process category, maximum number of *C. roseus* transcripts were assigned with the specific term, protein metabolism (13.17%) followed by response to stress (12.02%). GOSlim terms, nucleotide binding (9.81%), hydrolase activity (8.32%), transferase activity (8.03%) and protein binding (7.36%) were most represented under molecular function category. Among the cellular component category, nucleus (19%) followed by other cytoplasmic component (18.8%) were most represented (Fig. S5A). Based on COG (cluster of orthologous groups) classification, at least 33422 (56.43%) transcripts could be classified into 25 COG categories. Among the 25 COG categories, the cluster for general function prediction represented the largest group (7124; 21.31%), followed by post-translational modification, protein turnover, chaperones (3809; 11.40%) and signal transduction mechanisms (3380; 10.11%). In addition, 1941 (5.8%) of *C. roseus* transcripts were assigned into the cluster of unknown function (Fig. S5B).

To elucidate various biochemical pathways represented in the transcriptome, *C. roseus* transcripts were searched against Kyoto Encyclopedia of Genes and Genomes (KEGG) pathway database, which aids in studying the role of gene(s) in complex metabolic pathways. In total, 4436 genes (4738 transcripts) were found to be involved in one of the 318 different KEGG metabolic pathways. Few of the metabolic pathways represented with higher number of genes were, ribosome (117 genes), spliceosome (98 genes), biosynthesis of amino acids (96 genes), RNA transport (87 genes), purine metabolism (82 genes), carbon metabolism (80 genes), oxidative phosphorylation (73 genes), pyrimidine metabolism (71 genes) and protein processing in endoplasmic reticulum (71 genes). Along with plant hormone signal transduction (38 genes) pathway, we found genes involved in various alkaloid biosynthesis pathways also, like terpenoid backbone biosynthesis (26 genes), phenylalanine tyrosine and tryptophan biosynthesis (23 genes), ubiquinone and other terpenoid-quinone biosynthesis (17 genes), tryptophan metabolism (10 genes), tropane piperidine and pyridine alkaloid biosynthesis (eight genes), diterpenoid biosynthesis (11 genes), isoquinoline alkaloid biosynthesis (seven genes), sesquiterpenoid and triterpenoid biosynthesis (five genes), monoterpenoid biosynthesis (two genes) and indole alkaloid biosynthesis (one gene).

**Figure 1:**
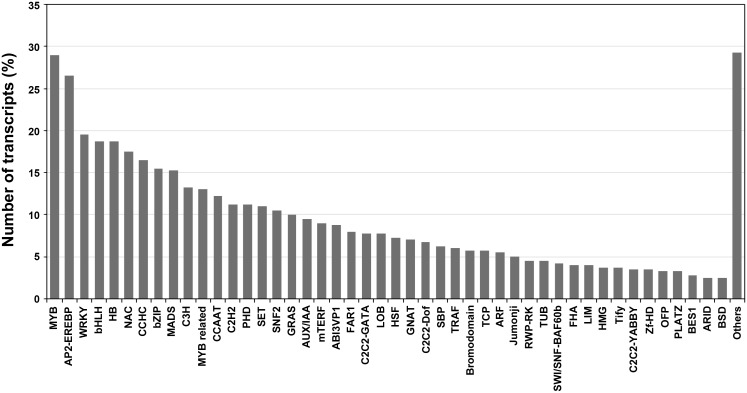
Number of transcripts representing different transcription factor families in *C. roseus* transcriptome.

We also identified TF-encoding genes in *C. roseus* transcriptome and found 1820 transcripts representing 73 TF families. Among the 73 families represented, the MYB-domain (116) family TFs were the most abundant followed by AP2-EREBP- (106), WRKY- (78), bHLH- (75) and HB- (75) domain TFs (Fig. 1). TFs play an important role in secondary metabolite and TIA accumulation [11], [17]. TFs known to be involved in TIA pathway like ORCA2, ORCA3, WRKY, MYC2 and zinc finger DNA-binding protein 1 and 2, were represented in the assembled transcriptome of *C. roseus*.

### Identification of simple sequence repeats

**Figure 2:**
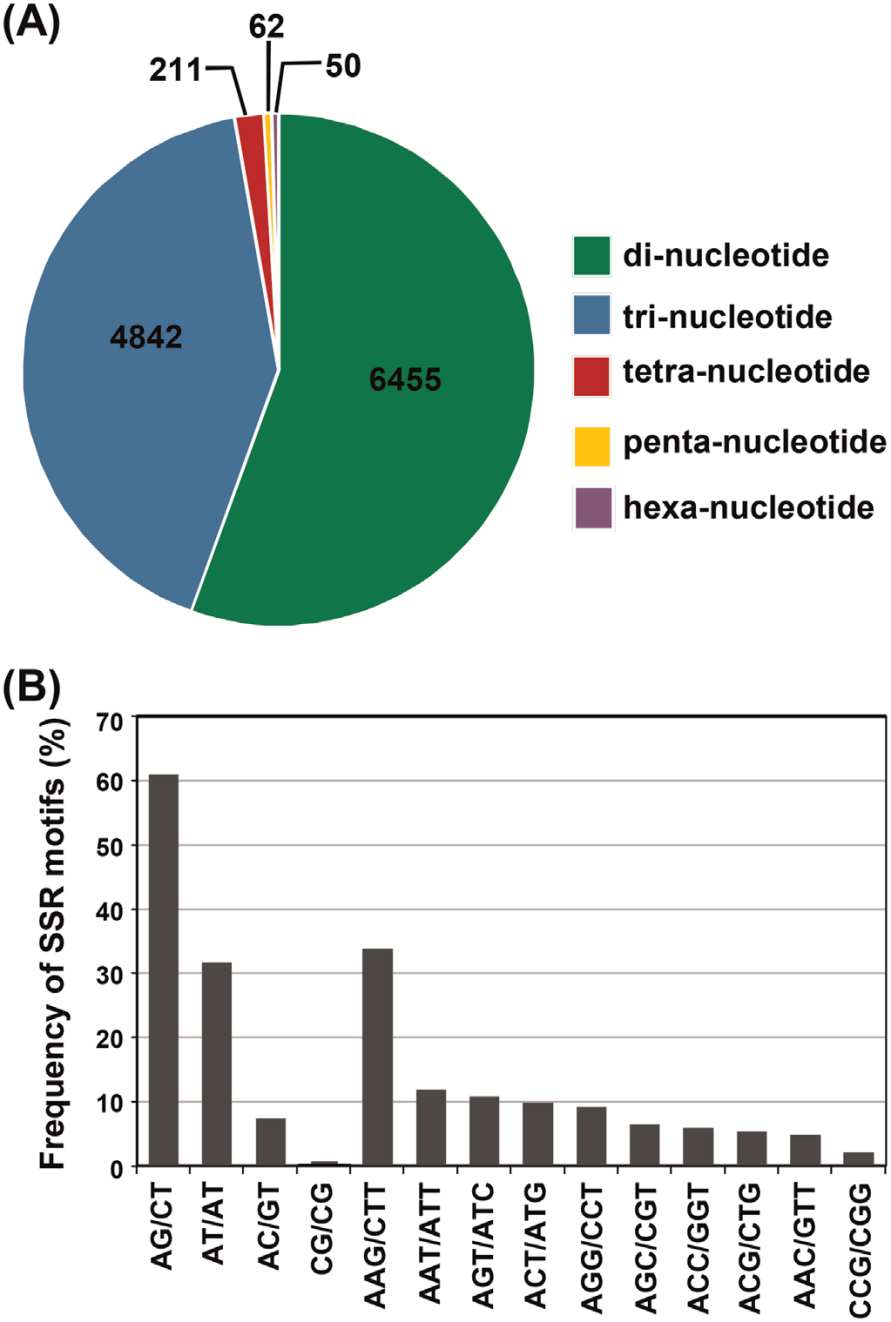
Identification of simple sequence repeats (SSRs) in *C. roseus* transcriptome. (A) Distribution of SSRs in different classes (B) Frequency of most common SSRs motifs is shown by bar graph.

Transcriptome resources have been harnessed for mining of SSRs in several plant species. EST-SSRs provide an insight on the density of SSRs in the transcribed region of genome and have higher rates of transferability across species [21]. *C. roseus* transcriptome was screened for SSRs using MISA search tool and a total of 11620 SSRs were identified in 8644 (14.6%) *C. roseus* transcripts. Among the identified EST-SSRs, di-nucleotide repeats were most represented (55.55%), followed by tri-nucleotide repeats with 41.67% (4842; Fig. 2A). Among di-nucleotide repeats, AG/CT showed highest occurrence (60.84%), followed by AT/AT (31.73%), AC/GT (7.41%) and CG/CG (0.03%). In case of tri-nucleotide repeats, occurrence of various motifs was uniform except for AAG/CTT, which showed highest frequency (34%) and CCG/CGG being the least abundant (2.15%) (Fig. 2B). Further, we developed a comprehensive SSR marker resource for *C. roseus* by designing forward and reverse primers from their flanking sequences. In total, we could design primers for 7158 (61.6%) SSR repeat-motifs identified, which can be used for the generation of functionally relevant markers in *C. roseus*. The complete list of SSRs identified in *C. roseus* along with primer sequences are available at Catharanthus Transcriptome Sequence web page.

### Differential gene expression and gene ontology enrichment analysis

**Figure 3:**
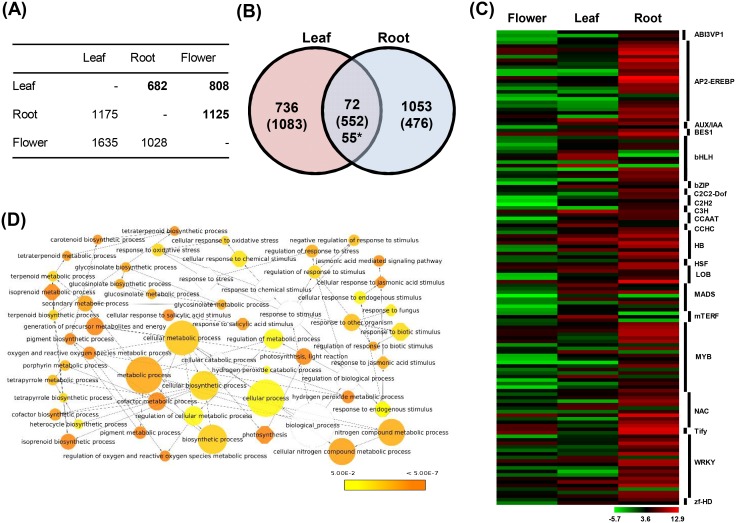
Differential expression analysis of *C. roseus* transcriptome. (A) Number of differentially expressed genes in different tissues in pairwise comparisons. Number of up-regulated genes are in bold, while down-regulated are in normal font. (B) Venn diagram showing number of up- and down-regulated (in parentheses) genes in leaf and root tissues as compared to flower. Asterisk represents genes up-regulated in one tissue and down-regulated in another tissue. (C) Heat-map showing expression patterns of differentially up-regulated TF encoding genes in different tissues. The scale at the bottom represents log_2_ fold change. (D) Graphical view showing GO terms associated with biological process enriched in up-regulated genes of leaf. The GO enrichment was performed using BiNGO. Node size is proportional to the number of genes in each category and shades represent the scale denoting significance level (white- no significant difference).

RNA-Seq has been considered to be the method of choice for differential gene expression studies at whole genome level [22], [23]. In total, approximately 87–90% short reads mapped onto *C. roseus* transcriptome and nearly 84–87% mapped uniquely (Table 2). DESeq package, was used to identify the genes differential expressed in different tissue samples [24]. We identified differentially expressed genes among different tissues of *C. roseus* via pairwise comparisons (Fig. 3A). Leaf and root being the primary site for alkaloid production, differential gene expression in these tissues as compared to flower was analyzed in more detail. A total of 2443 and 2153 genes were differentially expressed in leaf and root, respectively, as compared to flower tissue (Fig. 3A, B). In leaves, higher number of genes (1635) were down-regulated than 808 up-regulated genes, whereas in roots, there were nearly equal number of up- (1125) and down-regulated (1028) genes (Fig. 3A). Out of the total 4596 differentially expressed genes in leaf and roots, 679 were common. Among 679 genes commonly differentially expressed in roots and leaves, 72 and 552 genes were up- and down-regulated, respectively (Fig. 3B). Fifty five genes were found to be up-regulated in one tissue and down-regulated in another tissue. The genes related to photosynthesis were up-regulated in leaf, whereas genes annotated as DNA binding proteins (TFs), disease-resistant proteins and wound response proteins were up-regulated in roots. A heat-map of 1861 up-regulated genes in at least one tissue is represented in Fig. S6. Among 1861 up-regulated genes, at least 148 were found to encode for TFs, which are key regulatory proteins. TFs are known to play an important role in accumulation of secondary metabolites in plants [25]–[27]. Among 148 TFs exhibiting significant differential expression in leaf and root tissues, majority of the transcription factors belonging to AP2-EREBP, HB, MYB, NAC, Tify and WRKY families were up-regulated in root tissues (Fig. 3C).

**Table 2:** Mapping of non-redundant high-quality reads on *C. roseus* transcriptome.

Tissue samples	High quality reads	Total mapped reads (%)	Uniquely mapped reads (%)
**Leaf**	79025564	70974531 (89.81)	68635709 (86.85)
**Flower**	78728416	69955703 (88.86)	67774853 (86.09)
**Root**	72961718	63571877 (87.13)	61747179 (84.63)

We performed GO enrichment analysis to explore the major functional categories in up-regulated genes in leaves and roots. GO terms associated with various biological processes, such as metabolic process, nucleic acid metabolic process and cellular metabolic process were found to be enriched in up-regulated genes of leaf (Fig. S7A). Leaves being actively participating in photosynthesis, GO terms associated with photosynthetic process were also significantly enriched in leaves. Apart from these, biological process GO terms, like cellular response to jasmonic acid stimulus, ion homeostasis, carotenoid, isoprenoid and tertraterpenoid metabolic process were significantly enriched in genes up-regulated in leaf (Fig. 3D). Likewise, response to jasmonic acid stimulus was also significantly enriched in up-regulated genes of root. Thus, supporting the previous remarks that plant hormone jasmonic acid is one of the main drivers of TIA synthesis in *C. roseus* and plant secondary metabolism in general [7], [28]. Roots being an underground tissue is subjected to various biotic stresses present in the rhizosphere. GO terms like response to stress, response to biotic stimulus, defense response and response to fungus were significantly enriched in up-regulated root genes under biological process category. Regulation of transcription, cellular amino-acid derivative metabolic process, jasmonic acid biosynthesis, response to chemical stimulus, response to endogenous stimulus and response to salicylic acid were few other biological process GO terms, which were significantly enriched in up-regulated genes of roots (Fig. S7B).

### Expression profiling and validation of genes involved in TIA pathway

**Figure 4:**
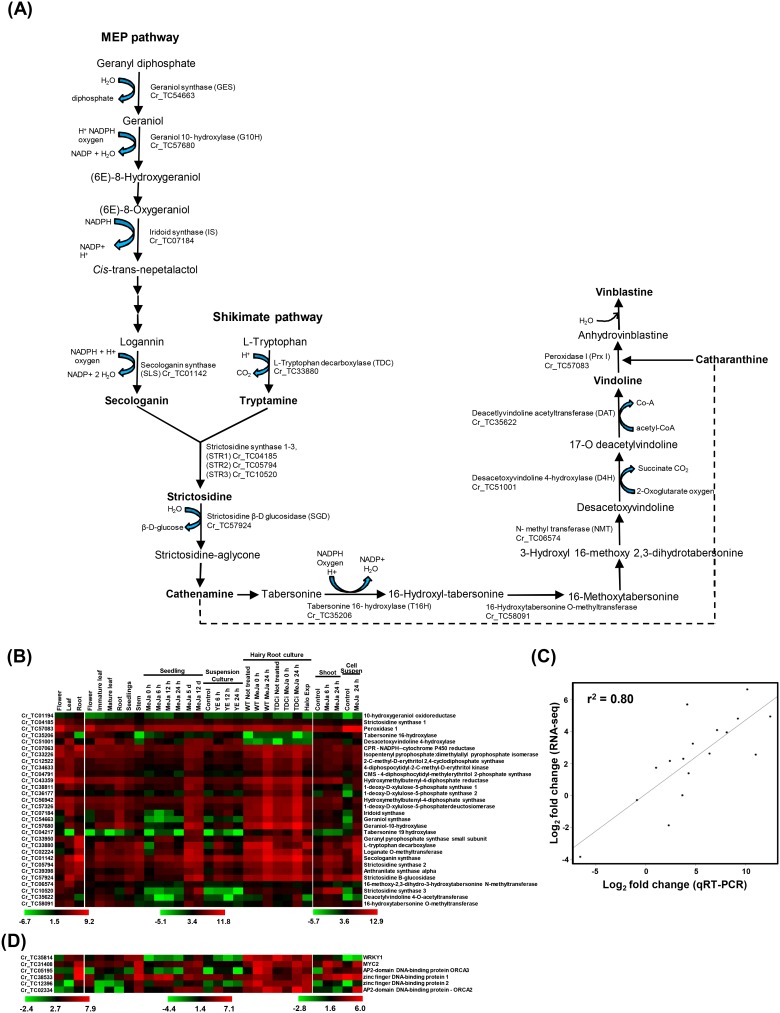
Expression patterns of transcripts involved in TIA biosynthesis. (A) Vindoline biosynthetic pathway showing important enzymes involved in different reactions. The IDs of *C. roseus* transcript encoding for the respective enzymes are also indicated. The important intermediates have been highlighted in bold font. (B) Heat-map showing expression patterns of TIA genes in different tissues and treatment. The scale at the bottom of each study represents log_2_ value of RPKM. Transcript IDs are given at left side and their putative annotation is on right side. (C) The correlation of gene expression results obtained from RNA-seq and real time RT-PCR analysis (D) Heat-map showing expression pattern of TF encoding genes in different tissues and treatment. The scale at the bottom of each study represents log_2_ value of RPKM. Transcript IDs are given at left side and their putative annotation is on right side.

For expression analysis, we mapped the short reads of individual sample from our study and previous study [16] onto our *C. roseus* transcriptome and analyzed expression profile using DESeq software. Complete expression analysis of genes is available at Catharanthus Transcriptome Sequence web page. This resource can be utilized by researchers to look for expression profile of their gene(s) of interest. *C. roseus* (L.) var. Prabal is well known for its high alkaloid content [29] and we were interested in expression analysis of important alkaloids (TIA) biosynthesis pathway genes. We used CathaCyc database [17] for the analysis of these pathways. The common precursor of TIAs, strictosidine, is the central intermediate formed by the coupling of tryptamine (shikimate pathway) and monoterpene secologanin (methyl erythritol phosphate pathway) as shown in Fig. 4A. The alkaloid, vinblastine, is synthesized by coupling of vindoline and catharanthine, both of which are obtained from branch-point intermediate cathenamine (Fig. 4A). We identified *C. roseus* transcripts encoding for most of the enzymes catalyzing different reactions involved in these pathways (Fig. 4A).

We identified 30 genes well-known to be involved in TIA biosynthetic pathways. The BLAST analysis showed that all of these genes were conserved in the sequenced genomes from Asterid clade (tomato and potato) and Arabidopsis at the protein level. However, only 17 (∼57%) and 22 (∼73%) of them exhibited significant similarity with annotated coding region sequences of Arabidopsis and Asterids (tomato and potato), respectively, at the nucleotide level. Further, we analyzed the expression of TIA biosynthetic pathway genes using RNA-Seq data in different tissue samples and treatments reported in our study and previous studies [16], [17]. As shown in the Fig. 4B, majority of the genes of TIA pathway were up-regulated in leaf and root tissues implying that these alkaloids are synthesized mainly in leaves and roots. Similar pattern of expression is also visible in developmental tissues used in the study by Gongora-Castillo et al. [16]. Both leaf and root tissues share more or less a common gene expression pattern for most TIA pathway genes, except for tabersonine 16-hydroxylase (Cr_TC35206) and deacetylvindoline 4-O-acetyltransferase (Cr_TC35622), which are highly down-regulated in roots and tabersonine 19-hydroxylase (Cr_TC04217), which is highly down-regulated in leaves (Fig. 4B). Apart from tabersonine 16-hydroxylase and deacetylvindoline 4-O-acetyltransferase, decreased expression of desacetoxyvindoline 4-hydroxylase was seen in root and hairy root cultures (Fig. 4B). Down-regulation of these enzymes in root tissues and hairy root cultures is in agreement with previous studies [8], [30], as they participate in terminal reactions for vindoline biosynthesis, which is restricted to aerial tissues. On the other hand, tabersonine 19-hydroxylase, which was found to be up-regulated in roots, further endorses previous finding that it helps to operate an alternate mechanism for tabersonine metabolism in roots by side-chain hydroxylation [31].

Gene expression analysis using RNA-seq data revealed that many of the genes involved in TIA pathway were differentially expressed in root and leaf tissues. To validate these findings, quantitative RT-PCR was performed for at least 10 genes of TIA pathway detected to be differentially expressed in root and leaf tissues. Real-time RT-PCR analysis revealed similar expression patterns of all the selected genes as observed in RNA-seq data. Moreover, the statistical analysis also showed a very good correspondence (correlation coefficient of 0.80) among the results of real time RT-PCR and RNA-seq data analysis as shown in Fig. 4C.

Suspension culture supplemented with yeast extract does not seem to be an attractive approach for TIA production due to lower gene expression as seen in gene expression profile (Fig. 4B). On the other hand, seedlings treated with MeJa showed increased expression, after exposure for longer duration i.e. 5 and 12 days. Expression of TIA genes was relatively higher in hairy root cultures, however it escalated when subjected to MeJa treatment (Fig. 4B). Expression profiling of TFs known to be involved in TIA pathway across different tissues and treatments revealed that their expression is highly up-regulated in hairy root culture, stem and root (Fig. 4D). Similar to earlier observations, there was a growth related decrease in TIA transcripts in *C. roseus* and accumulation of bisindole alkaloid content depends on tissue maturity [8], [30], [32], we also found that the expression of TIA genes diminished in the mature leaf tissue.

## Discussion

*C. roseus* is widely known for its pharmaceutical potential and has become one of the extensively studied medicinal plants. It is considered to be single biological source of the anti-cancer compounds, vinblastine and vincristine [2], [33]. Although many good efforts have been made to elucidate the complete pathway of TIA biosynthesis, but few complex steps and intermediate compounds are still unknown. Transcriptome studies, with the advent of next generation sequencing technologies, can help addressing few of these problems via gene discovery. Here, we performed high-throughput sequencing of transcriptome from different tissues of *C. roseus* and used short read assembly tools (Velvet, Oases and ABySS) for *de novo* assembly optimization. A two-step strategy involving merging of best k-mer assembly (from out data) with earlier reported MPGR assembly [16] was employed to obtain a robust *C. roseus* transcriptome.

Based upon various parameters [18], assembly generated from Oases at k-mer length of 57 taking NR short reads was considered to be the best. This is in agreement with a previous study by Ghangal et al. [34] who also reported that assembly generated from NR reads was better than total reads. Merging of best k-mer assembly (NR-Oases-k-57) with MPGR assembly using TGICL further improved the assembly assessment parameters, such as N50 length (2115 bp), average transcript length (1283 bp) and sequence similarity with closely related species. As sequence similarity also marks the completeness of the transcriptome, 77% of the known full-length *C. roseus* proteins were found to be present in our *C. roseus* transcriptome. We also found all the previously reported [17] genes involved in TIA bio-synthesis (30 genes) represented in our assembled transcriptome. The presence of already reported important alkaloid biosynthetic genes and full-length proteins marks the quality of *C. roseus* transcriptome. Moreover, BLAST analysis with earlier transcriptome sequences of *C. roseus* [16], [17] and other related plant proteome and transcriptome sequences revealed a better transcriptome assembly presented in our study.

For comprehensive annotation, *C. roseus* transcriptome was subjected to similarity search against various known protein databases. We were able to annotate about 65% of *C. roseus* transcripts. Recently discovered genes encoding geraniol synthase (GES) [35] and iridoid synthase (IS) [36], known to be involved in biosynthesis of secologanin (a monoterpenoid alkaloid) from geranyl pyrophosphate were also present in our *C. roseus* transcriptome. Overall, more than 56% of transcripts were classified into 25 COG categories, which is quite higher than other studies [37]–[39]. We found the category “general function prediction” to be the most represented in COG classification accounting for its need for basic physiological and metabolic functions. The GC content of *C. roseus* transcriptome was found to be very much similar to other dicot plants. Our results concord with earlier findings that there is only marginal variation in average GC content between dicots like Arabidopsis, soybean, tomato, potato, pea and tobacco [40].

Many studies have been undertaken to characterize and differentiate different *C. roseus* cultivars using various molecular markers, such as AFLP [41], [42], RAPD [41], [43], ISSR [42] and SSRs [44]–[46]. Microsatellites are co-dominant molecular markers used for marker-assisted selection studies and their identification from high-throughput transcriptome studies have been reported in large number of plant species. A total of 11620 SSRs of 2–6 nucleotides were predicted in *C. roseus* transcripts with di-nucleotides repeats being most abundant followed by tri-nucleotide repeat. This is in accordance with previous studies on *C. roseus*, who also observed more di-nucleotide repeats than tri-nucleotide repeats in the EST datasets [46]. The availability of a large number of SSRs with primer sequences can help large-scale genotyping studies for various applications. Existence of genetic diversity in *C. roseus* have been demonstrated by developing STMS markers [44], [45]. Thus, availability of transcriptome for screening of SSRs hold an immense potential for high-throughput genotyping applications in *C. roseus*.

TFs are key regulators that can alter the gene expression of several target genes, thereby can regulate metabolic flux. Members of some TF families, such as MYB, AP2-EREBP, WRKY, MYB-related and bHLH, are known to regulate secondary metabolism in plants [47]–[49]. Members of the plant-specific AP2-EREBP TF family, namely octadecanoid-derivative responsive Catharanthus AP2-domain protein (ORCA2 and ORCA3), which are known to activate expression of several genes (enzymes) involved in TIA biosynthesis, were identified in our *C. roseus* transcriptome. Recently, Suttipanta et al. [48] characterized CrWRKY1 and reported its involvement in the transcriptional regulation of TIA pathway in *C. roseus*. Apart from AP2-domain and WRKY proteins, we also found MYC2, zinc-finger DNA binding protein 1 and 2 in the *C. roseus* transcriptome, which were reported by Van Moerkercke et al. [17] to be involved in regulation of TIA biosynthesis.

Digital expression profiling, a powerful and efficient approach for *in-silico* analysis of gene expression, was employed to determine the expression of genes involved in TIA biosynthesis. When compared with flower, genes involved in TIA biosynthesis were highly active in leaf and root tissues. Except few genes, majority of the TIA pathway genes were up-regulated in leaves and roots, which are the prime source of anticancer and antihypertensive alkaloids, respectively. Our study further confirms previous findings that some of the enzymes involved in late reactions of vindoline biosynthesis are not expressed in cell cultures or in tissues unable to produce vindoline [50], [51]. As reported earlier by Goklany et al. [52], TIA pathway genes are up-regulated in hairy root cultures elicited with MeJa [9], [53], we also observed an increase in gene expression of TIA pathway genes in MeJa induced hairy root cultures. Elicitor, like MeJa are compounds, which induce plant stress response and thereby increasing gene expression and alkaloid biosynthesis. Looking at overall digital expression profiling of TIA pathway genes, we conclude that expression of genes are dependent on plant maturity and are highly expressed in MeJa-elicited hairy root cultures. Nearly equal number of genes were differentially expressed in leaf and roots. Enrichment of GO terms, performed on differentially expressed genes showed that photosynthesis related genes were up-regulated in leaves. GO terms, like jasmonic acid biosynthesis, response to jasmonic acid stimulus, isoprenoid and tetraprenoid metabolic process were also enriched in differentially expressed genes. This further adds on to the findings that MeJa induces expression of TIA pathway genes.

In conclusion, we assembled and annotated *C. roseus* transcriptome. Many transcripts harboring microsatellite repeats were identified, which can be used for marker-assisted breeding in *C. roseus*. Differential gene expression and GO enrichment analyses revealed the enrichment of genes involved in secondary metabolite production in leaf tissues, which is the prime source of bisindole alkaloids. Further, expression profiling of TIA genes determined that vindoline exclusively accumulates in aerial tissue of *C. roseus* and exposure to MeJa increases its production. However, deeper understanding of regulatory network governing TIA biosynthesis could help in successful metabolic engineering of alkaloid biosynthesis. The transcriptome resource generated in this study can facilitate understanding of regulatory and metabolic pathways underlying the biosynthesis of alkaloids.

## Materials and Methods

### RNA isolation, sequencing and quality filtering

Leaf, root and flower tissues of *C. roseus* L. var. Prabal were harvested from the adult plants grown in field. The tissues were harvested from the plants grown under natural environmental conditions in the experimentation field (28°31′55.3′′N 77°09′54.9′′E) of the National Institute of Plant Genome Research, New Delhi. The field experiments conducted in this study did not involve endangered or protected species and no specific permission was required for these location/activities. The tissues were snap frozen in liquid nitrogen and stored in −80°C until further use. RNA was isolated from tissue samples using TRI reagent (Sigma Life Science, USA). Quantity and quality of RNA samples were measured using Nanodrop (Thermo Fisher Scientific) and Agilent Bioanalyzer (Agilent technologies, Singapore). Sequencing was performed using HiSeq 2000 platform generating paired-end reads of 100 bp length. Stringent quality check was performed on short read datasets by using NGS QC Toolkit v2.3 [54] to remove the low quality reads and those having primer/adaptor contamination. Duplicate reads from the dataset were removed using CLC Genomic Workbench (v4.7.2, http://maq.sourceforge.net/index.shtml) to obtain NR dataset.

### *De novo* short read assembly and validation

*De novo* transcriptome assembly was performed by using three commonly used short read assemblers, Velvet (v1.2.01) [55], Oases (v0.2.04) [56] and ABySS (v1.2.6) [57]. All the three assemblers used in this study were run at different k-mer lengths, ranging from 31–95. We employed a two-step approach, the best k-mer assembly obtained from different assemblers was merged with MPGR *C. roseus* transcriptome [16] and subjected to second round of assembly using TGICL (v2.0) [58] with minimum and maximum overlap length of 40 and 90, respectively. Various assembly parameters kept in consideration for marking the best assembly has been described previously by Garg et al. [18]. GC content analysis was done using in-house perl script. To validate the quality of assembled transcriptome, we performed simple and reciprocal BLAST searches at an *E*-value cut-off of ≤1e-05 for identification of best significant match. The proteome sequences of tomato, potato, cucumber, grapevine and Arabidopsis were downloaded from phytozome v9.1 (www.phytozome.net) and transcriptome sequences of alkaloid producing plants (*A. belladonna*, *C. acuminata*, *C. sativa*, *R. serpentine*, *R. officinalis* and *V. officinalis*) were downloaded from Medicinal plant genomics resource (www.medicinalplantgenomics.msu.edu).

### Functional annotation

One of the most common approach for annotating transcriptome assembly is similarity search via BLAST. *C. roseus* transcripts were searched against TAIR10 proteome, Uniref90, Uniref100 and non-redundant protein (NCBI-nr) data sets at an *E*-value cut-off of ≤1e-05 for identification of best significant match. GOSlim terms for molecular function, biological process and cellular component were assigned to each *C. roseus* transcripts on the basis of their best match Arabidopsis protein. Similarity search against COG database classified *C. roseus* transcripts among different categories of COG classification system. To look for the genes involved in various pathways, assignment of KEGG Orthology (KO) terms and KEGG pathway construction was performed using KAAS (KEGG Automatic Annotation Server) [59] at default parameters.

### Read mapping and gene expression analysis

For gene expression analysis, high-quality short reads were mapped on to *C. roseus* transcriptome assembly using RNA-seq analysis utility of CLC Genomics Workbench. A maximum of two mismatches were permitted for alignments. Unique read counts for each tissue sample were normalized by calculating the read per kilo-base per million (RPKM) for each transcript. DESeq (v1.10.1) [24], a software of R package, was used for differential gene expression analysis. It measures gene expression based on the negative binomial distribution with variance and mean linked by local regression. We calculated the size factor for each sample for normalization of read count data using DESeq. A p-value cut-off of ≤0.05 and at least two-fold change in gene expression was used to identify differentially expressed genes. RPKM values were log_2_ transformed and heat-map showing expression profiles for genes involved in TIA pathway were generated using MultiExperiment Viewer (MeV, v4.8). Hierarchical clustering was performed using Pearson correlation metrics and average linkage rule using MeV.

### Real-time PCR analysis

For real-time PCR analysis, gene-specific primers (Table S4) were designed using Primer Express (v3.0) software (Applied Biosystems, USA). Actin was used as an internal control. At least three independent biological replicates with three technical replicates of each biological replicate for each tissue sample were used for analysis. Real-time PCR reactions were carried out essentially following the protocol described previously [60]. The correlation between expression profiles of selected genes obtained from real-time RT-PCR and RNA-seq data analysis was determined in R program.

### Identification of SSR and transcription factors

*C. roseus* transcriptome was screened for the presence of microsatellites (SSRs) using MISA [61]. The number of repeating units considered in this study was, six for di-nucleotides, and five for tri-, tetra-, penta- and hexa-nucleotides. We did not consider mono-nucleotide repeats in this study. Primers for all the identified SSRs were designed using BatchPrimer3 v1.0 (probes.pw.usda.gov/batchprimer3). TFs encoding *C. roseus* transcripts were identified based on the Hidden Markov Model (HMM) profile search of conserved domain present in each TF family as described earlier [18].

### GO enrichment analysis

For GO enrichment analysis, similarity search (BLASTX) was carried out against Arabidopsis proteome and the best hit corresponding to each *C. roseus* transcripts was identified. GO enrichment of different sets of genes was performed using BiNGO tool [62] as described previously [63].

## Supporting Information

Figure S1 **Number of** ***C. roseus*** **transcripts showing significant similarity with proteome/transcriptome sequences of closely related/alkaloid producing plants.**
(PDF)

Figure S2 **Reciprocal BLAST analysis of** ***C. roseus*** **transcripts showing number of orthologous genes in closely related plant species.**
(PDF)

Figure S3 **Length distribution of transcripts in the** ***C. roseus*** **transcriptome.**
(PDF)

Figure S4 **GC content distribution in the** ***C. roseus*** **and** ***A. thaliana*** **transcripts.**
(PDF)

Figure S5 **Functional annotation of** ***C. roseus*** **transcripts.** (A) GOSlim term assignment to the *C. roseus* transcripts in different categories of biological process, molecular function and cellular component. (B) COG function classification of *C. roseus* transcripts.
(PDF)

Figure S6 **Heat-map showing expression patterns of differentially up-regulated genes in different tissues of** ***C. roseus*** **analyzed in this study.** The scale at the bottom represents log_2_ fold change.
(PDF)

Figure S7 **Graphical view showing biological process gene ontology term enrichment in up-regulated genes in (A) leaf and (B) root.** The GO enrichment was performed using BiNGO. Node size is proportional to the number of genes in each category and shades represent the scale denoting significance level (white- no significant difference).
(PDF)

Table S1 Quality control and duplicate read removal statistics of *C. roseus* libraries.
(PDF)

Table S2 *De novo* assembly statistics by different assemblers at different k-mer length using total high-quality reads (a) Velvet (b) Oases (c) ABySS.
(PDF)

Table S3 *De novo* assembly statistics by different assemblers at different k-mer length using NR high-quality reads (a) Velvet (b) Oases (c) ABySS.
(PDF)

Table S4 Primer sequences used for real-time PCR analysis.
(PDF)
