# Supplementary material for: Transcriptome Analysis of Catharanthus roseus for Gene Discovery and Expression Profiling
Source: PLoS One. 2014 Jul 29;9(7):e103583. doi: 10.1371/journal.pone.0103583 (PMC4114786; doi:10.1371/journal.pone.0103583)

Fig S1. Number of *C. roseus* transcripts showing significant similarity with proteome/transcriptome sequences of closely related/alkaloid producing plants.

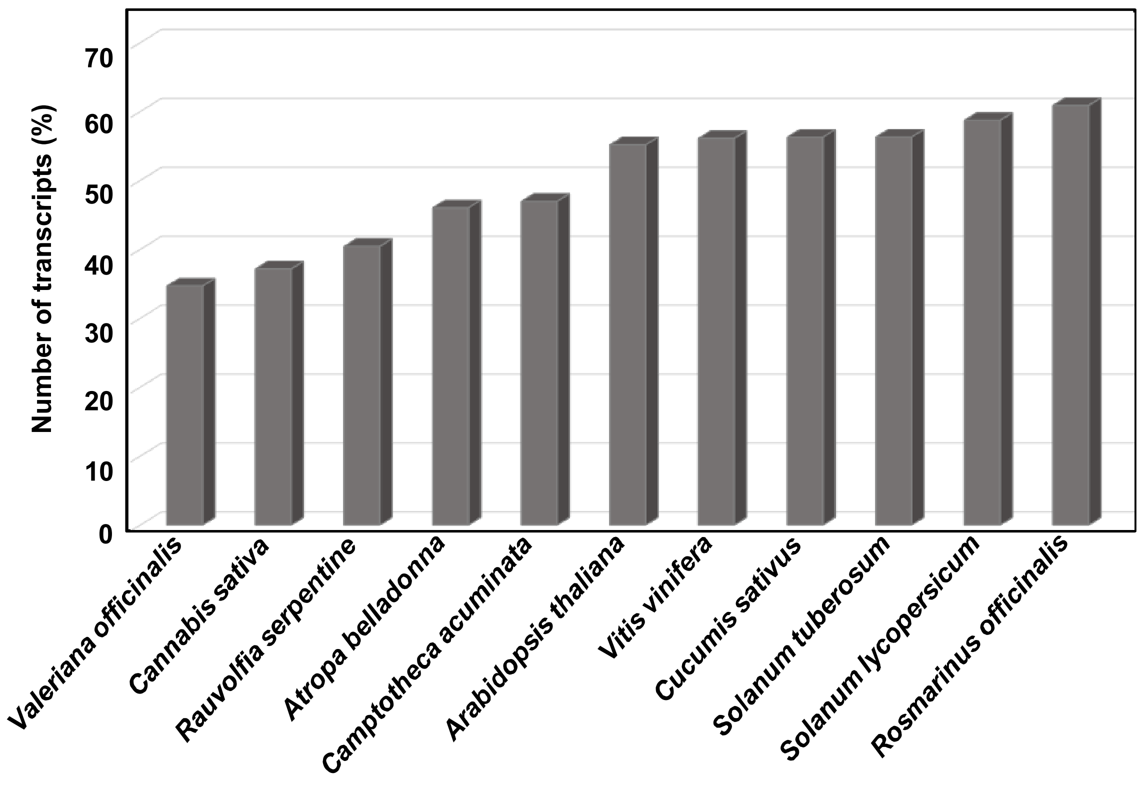

Supplement: Figure S1 — Number of C. roseus transcripts showing significant similarity with proteome/transcriptome sequences of closely related/alkaloid producing plants. (PDF) [file pone.0103583.s001.pdf]
