# Supplementary material for: Transcriptome Analysis of Catharanthus roseus for Gene Discovery and Expression Profiling
Source: PLoS One. 2014 Jul 29;9(7):e103583. doi: 10.1371/journal.pone.0103583 (PMC4114786; doi:10.1371/journal.pone.0103583)

Fig S2. Reciprocal BLAST analysis of *C. roseus* showing number of orthologous genes in closely related plant species.

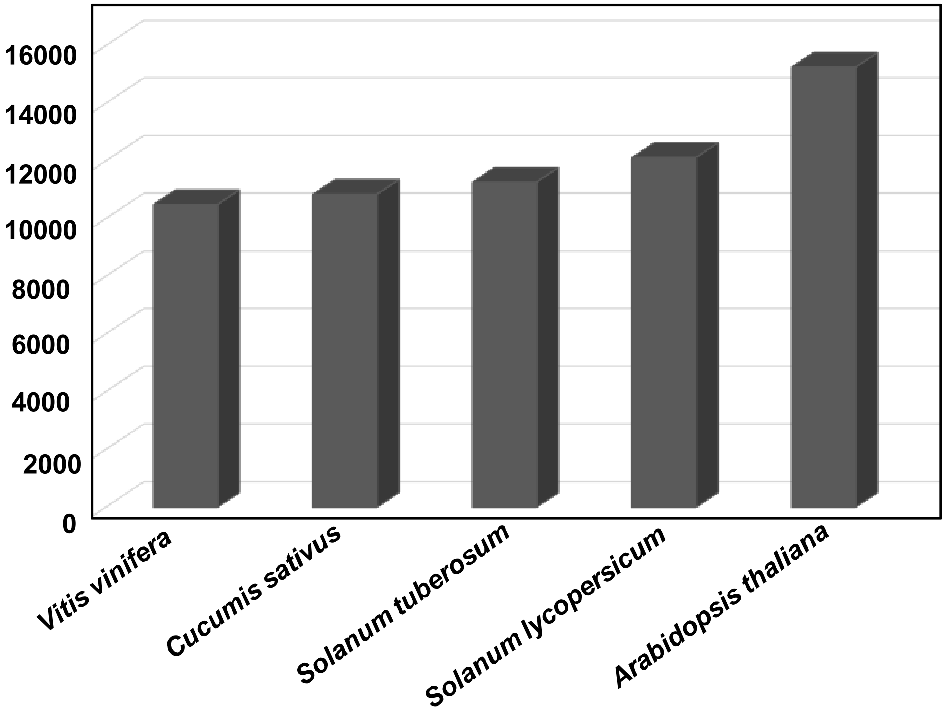

Supplement: Figure S2 — Reciprocal BLAST analysis of C. roseus transcripts showing number of orthologous genes in closely related plant species. (PDF) [file pone.0103583.s002.pdf]
