# Supplementary figures and images for: Transcriptome Analysis of Catharanthus roseus for Gene Discovery and Expression Profiling
Source: PLoS One. 2014 Jul 29;9(7):e103583. doi: 10.1371/journal.pone.0103583 (PMC4114786; doi:10.1371/journal.pone.0103583)

**Fig S3.** Length distribution of transcripts in the *C. roseus* transcriptome.

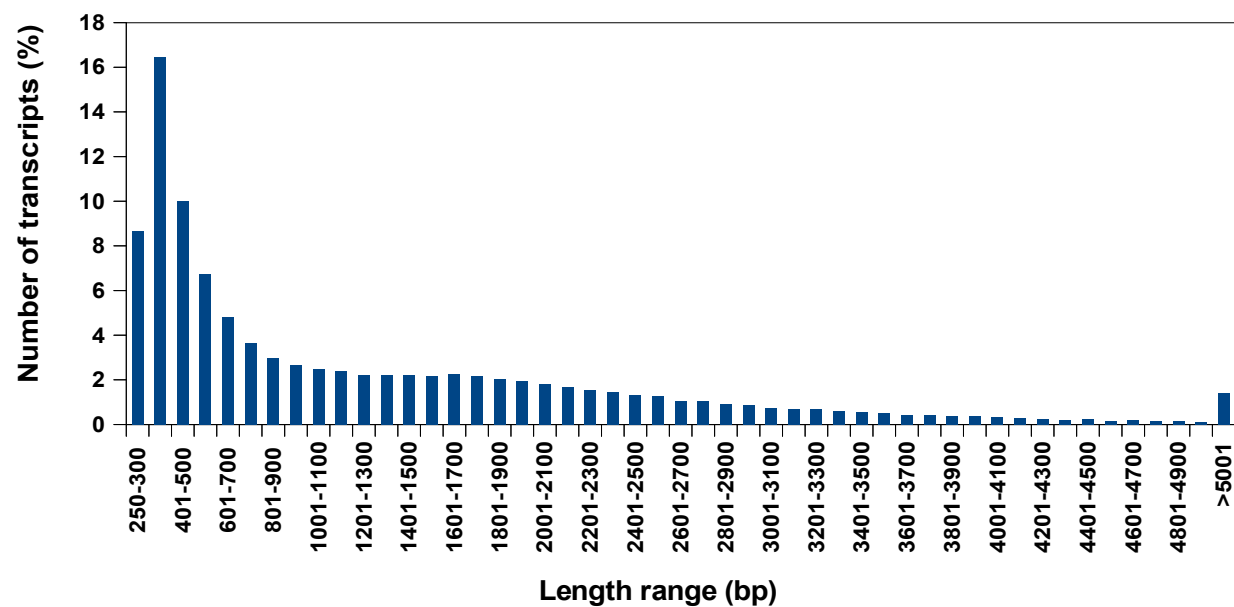

Supplement: Figure S3 — Length distribution of transcripts in the C. roseus transcriptome. (PDF) [file pone.0103583.s003.pdf]

**Fig S4.** GC content distribution in the *C. roseus* and *A. thaliana* transcripts.

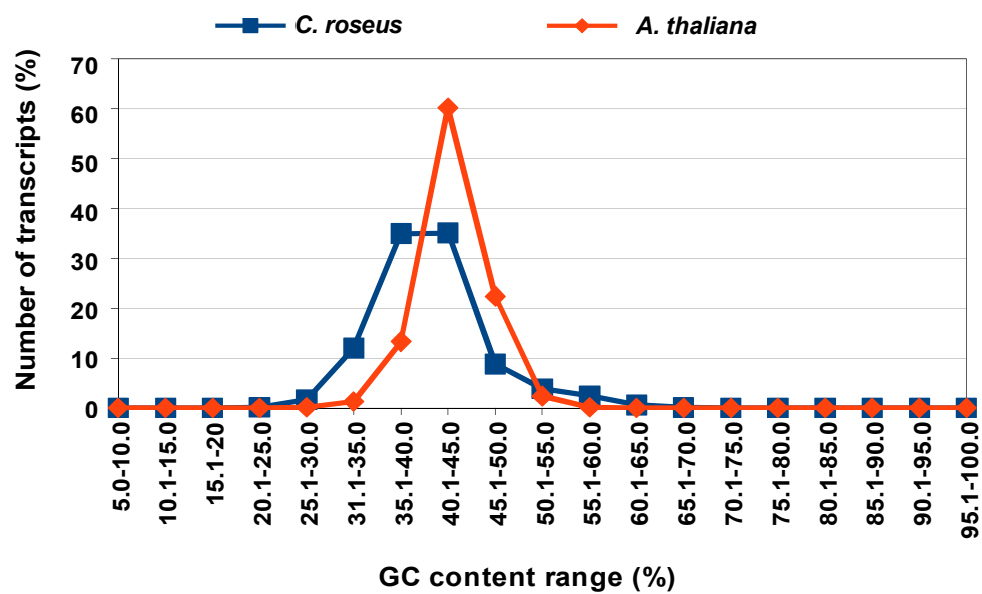

Supplement: Figure S4 — GC content distribution in the C. roseus and A. thaliana transcripts. (PDF) [file pone.0103583.s004.pdf]
