# Supplementary material for: Transcriptome Analysis of Catharanthus roseus for Gene Discovery and Expression Profiling
Source: PLoS One. 2014 Jul 29;9(7):e103583. doi: 10.1371/journal.pone.0103583 (PMC4114786; doi:10.1371/journal.pone.0103583)
Supplement: Figure S5 — Functional annotation of C. roseus transcripts. (A) GOSlim term assignment to the C. roseus transcripts in different categories of biological process, molecular function and cellular component. (B) COG function classification of C. roseus transcripts. (PDF) [file pone.0103583.s005.pdf]

Fig S5. Functional annotation of *C. roseus* transcripts. (A) GOSlim term assignment to the *C. roseus* transcripts in different categories of biological process, molecular function and cellular component. (B) COG function classification of *C. roseus* transcripts.

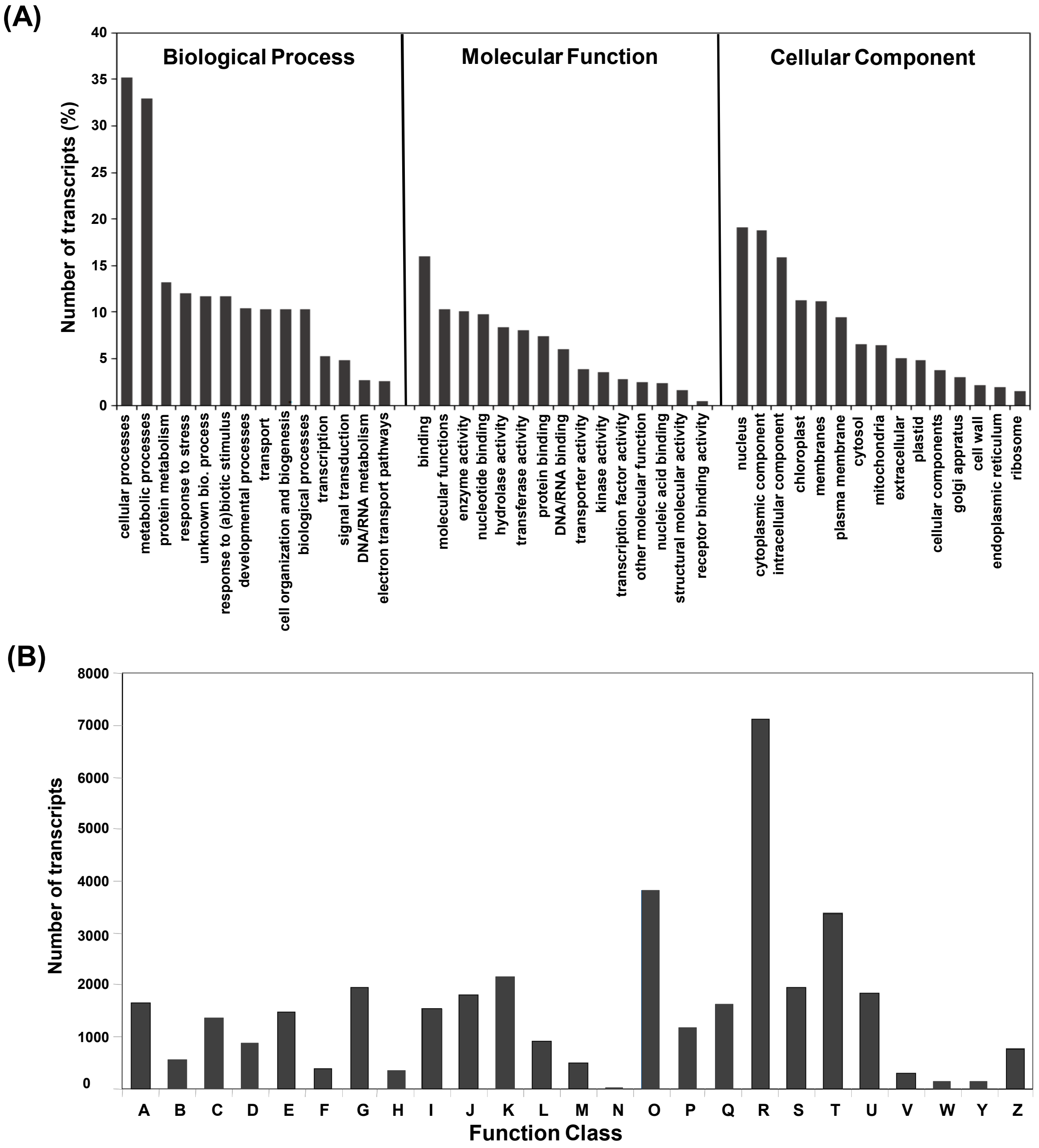

A RNA processing and modification
B Chromatin structure and dynamics
C Energy production and conversion
D Cell cycle control, cell division, chromosome partitioning
E Amino acid transport and metabolism
F Nucleotide transport and metabolism
G Carbohydrate transport and metabolism
H Coenzyme transport and metabolism
I Lipid transport and metabolism
J Translation, ribosomal structure and biogenesis
K Transcription
L Replication, recombination and repair
M Cell wall/membrane/envelope biogenesis
N Cell motility
O Post translational modification, protein turnover, chaperones
P Inorganic ion transport and metabolism
Q Secondary metabolite biosynthesis, transport and catabolism
R General function prediction only
S Function unknown
T Signal Transduction mechanisms
U Intracellular trafficking, secretion and vesicular transport
V Defense mechanisms
W Extracellular structures
Y Nuclear structures
Z Cytoskeleton
