# Supplementary material for: Transcriptome Analysis of Catharanthus roseus for Gene Discovery and Expression Profiling
Source: PLoS One. 2014 Jul 29;9(7):e103583. doi: 10.1371/journal.pone.0103583 (PMC4114786; doi:10.1371/journal.pone.0103583)

Fig. S6. Heat map showing expression patterns of differentially up-regulated genes in different tissues of *C. roseus* analyzed in this study. The scale at the bottom represents $\log_2$ fold change.

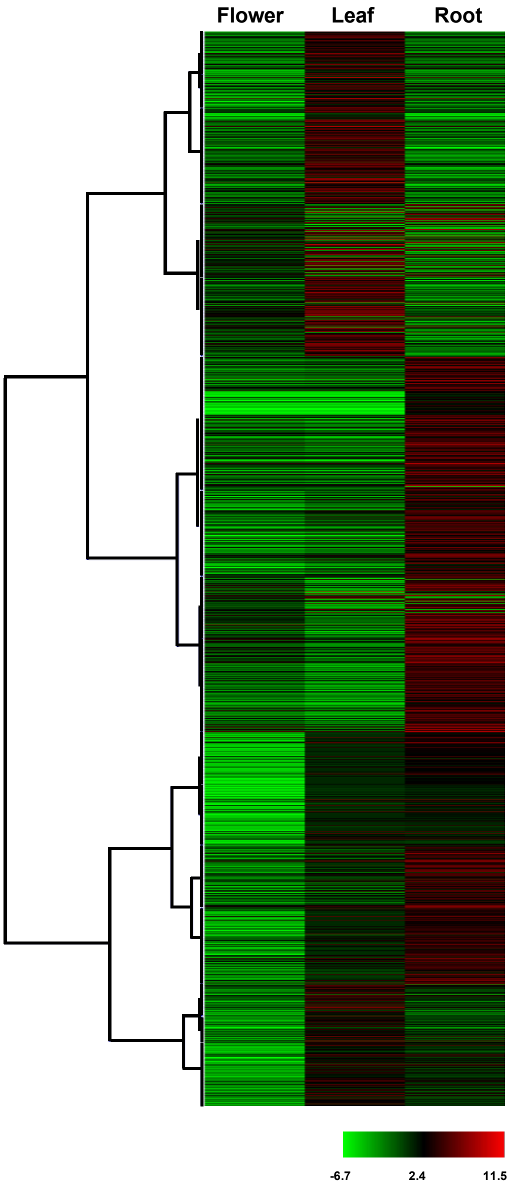

Supplement: Figure S6 — Heat-map showing expression patterns of differentially up-regulated genes in different tissues of C. roseus analyzed in this study. The scale at the bottom represents log2 fold change. (PDF) [file pone.0103583.s006.pdf]
