# Supplementary material for: Transcriptome Analysis of Catharanthus roseus for Gene Discovery and Expression Profiling
Source: PLoS One. 2014 Jul 29;9(7):e103583. doi: 10.1371/journal.pone.0103583 (PMC4114786; doi:10.1371/journal.pone.0103583)
Supplement: Table S1 — Quality control and duplicate read removal statistics of C. roseus libraries. (PDF) [file pone.0103583.s008.pdf]

Table S1: Quality control and duplicate read removal statistics of *C. roseus* libraries.

Tissue samples	Total reads	High quality reads[1] (Total reads)	High quality reads[2] (Non-redundant reads)
**Leaf**	119566382	118316318	79025564
**Flower**	116361304	115092434	78728416
**Root**	111305576	109975332	72961718
**Total**	347233262	343384084	230715698

[1]High quality reads obtained after filtering using NGS QC tool kit.
[2]High quality non-redundant reads (after removal of duplicate reads)
