# Supplementary material for: Transcriptome Analysis of Catharanthus roseus for Gene Discovery and Expression Profiling
Source: PLoS One. 2014 Jul 29;9(7):e103583. doi: 10.1371/journal.pone.0103583 (PMC4114786; doi:10.1371/journal.pone.0103583)
Supplement: Table S2 — De novo assembly statistics by different assemblers at different k-mer length using total high-quality reads (a) Velvet (b) Oases (c) ABySS. (PDF) [file pone.0103583.s009.pdf]

Table S2: *De novo* assembly statistics by different assemblers at different k-mer length using total high-quality reads

(A) Velvet

	K_35	K_39	K_43	K_51	K_55	K_57	K_59	K_61	K_65	K_69	K_75	K_89	K_93	K_95
**Number of contigs**	483493	471545	442626	445123	374482	344569	317569	290418	237720	197921	146582	57353	39010	30453
**Total size (Mb)**	92.69	93.19	91.59	93.53	88.00	84.91	81.97	78.87	72.26	66.29	57.08	34.02	25.38	18.99
**Minimum length (bp)**	100	100	100	101	109	113	117	121	129	137	149	177	185	189
**Maximum length (bp)**	2890	3060	3064	5366	6542	8657	8358	11923	11924	9511	11337	13745	10893	7142
**Average length (bp)**	191.71	197.64	206.93	210.14	235.00	246.44	258.12	271.58	304.0	334.95	389.46	593.20	650.73	623.82
**N50 length (bp)**	185	193	205	238	272	287	303	321	362	403	472	799	848	745

(B) Oases

	K_31	K_35	K_39	K_43	K_51	K_55	K_57	K_59	K_61	K_65
**Number of contigs**	296098	269768	241269	213235	163979	146525	138587	133289	128716	118132
**Total size (Mb)**	178.22	177.30	179.99	151.09	176.93	179.19	175.48	170.68	165.01	151.25
**Minimum length (bp)**	100	100	100	100	100	100	100	100	100	100
**Maximum length (bp)**	35537	22926	18370	23799	19103	17112	17881	17112	17071	17034
**Average length (bp)**	601.92	657.25	746.03	849.29	1079.03	1222.99	839.00	866.00	1281.99	1280.41
**N50 length (bp)**	1238	1347	1537	1706	1867	2133	2194	2190	2161	2099

(C) AByS S

	K_63	K_65	K_77	K_83	K_85	K_87	K_89	K_93
**Number of contigs**	349522	330402	216379	166405	148636	133650	115571	83324
**Total size (Mb)**	136.59	134.29	107.42	88.58	80.79	72.78	63.95	45.17
**Minimum length (bp)**	100	100	100	100	100	100	100	100
**Maximum length (bp)**	15324	15324	14839	15578	15524	15524	15389	15611
**Average length (bp)**	390.79	406.47	496.49	532.35	543.60	544.56	553.42	542.19
**N50 length (bp)**	750	766	925	1024	1061	1087	1127	1155
