# Supplementary material for: Transcriptome Analysis of Catharanthus roseus for Gene Discovery and Expression Profiling
Source: PLoS One. 2014 Jul 29;9(7):e103583. doi: 10.1371/journal.pone.0103583 (PMC4114786; doi:10.1371/journal.pone.0103583)
Supplement: Table S3 — De novo assembly statistics by different assemblers at different k-mer length using NR high-quality reads (a) Velvet (b) Oases (c) ABySS. (PDF) [file pone.0103583.s010.pdf]

Table S3. ***De novo*** **assembly statistics by different assemblers at different k-mer length using NR high-quality reads.**

(A) Velvet

	K_31	K_35	K_39	K_43	K_51	K_55	K_57	K_59	K_61	K_65	K_69	K_75	K_89	K_93	K_95
**Number of contigs**	500170	480499	468253	439096	408330	343218	314713	287008	260789	213855	177620	134574	56021	39276	30848
**Total size (Mb)**	92.45	92.36	92.86	91.30	90.00	84.79	81.77	78.67	75.56	69.49	63.83	55.61	34.00	25.62	19.22
**Minimum length (bp)**	100	100	100	100	101	109	113	117	121	129	137	149	177	185	189
**Maximum length (bp)**	2745	2889	3064	3064	5366	5784	8657	6927	11923	11924	9511	11337	15591	10913	7142
**Average length (bp)**	184.85	192.22	198.33	207.94	220.42	247.05	259.85	274.12	289.76	324.97	359.41	413.27	606.93	652.32	623.32
**N50 length (bp)**	177	186	194	207	250	286	303	321	340	383	426	494	809	841	738

(B) Oases

	K_31	K_35	K_39	K_43	K_51	K_55	K_57	K_59	K_61	K_65	K_69	K_75
**Number of contigs**	56982	56084	55560	55493	58313	54064	53017	53110	52993	52499	51197	47859
**Total size (Mb)**	54.06	53.42	54.09	54.40	55.77	52.77	51.86	51.35	50.90	49.74	48.04	44.62
**Minimum length (bp)**	100	100	100	100	101	105	107	109	111	115	119	125
**Maximum length (bp)**	17314	17326	16362	17052	15948	17112	17112	17112	17071	17034	15601	15441
**Average length (bp)**	948.78	952.67	973.7	980.37	956.41	976.11	978.19	966.9	960.62	947.53	938.45	932.52
**N50 length (bp)**	2061	1995	1975	1934	1718	1881	1911	1879	1851	1773	1715	1648

(C) AByss

	K_63	K_65	K_77	K_83	K_85	K_87	K_89	K_93
**Number of contigs**	145910	138938	110435	87062	78269	71190	69034	54820
**Total size (Mb)**	91.36	89.48	80.12	68.04	62.92	57.62	53.68	37.49
**Minimum length (bp)**	100	100	100	100	100	100	100	100
**Maximum length (bp)**	15473	15697	15577	15581	15524	15524	15633	15611
**Average length (bp)**	626.18	644.08	725.53	781.55	803.97	809.47	777.63	683.94
**N50 length (bp)**	1202	1218	1279	1350	1387	1400	1379	1298
