# Supplementary material for: Transcriptome Analysis of Catharanthus roseus for Gene Discovery and Expression Profiling
Source: PLoS One. 2014 Jul 29;9(7):e103583. doi: 10.1371/journal.pone.0103583 (PMC4114786; doi:10.1371/journal.pone.0103583)
Supplement: Table S4 — Primer sequences used for real-time PCR analysis. (PDF) [file pone.0103583.s011.pdf]

Table S4. Primer sequences used for real-time PCR analysis.

Transcript ID	Forward Primer	Reverse Primer
**Cr_TC35622**	TGGAGTACTGGAAATGGCAAAGA	CCACGGTAGCAGGGAAATCA
**Cr_TC01142**	GCAAGAGCGAGCTAGGGAAGA	TTAAGCGTTCCAATTCAGGTTTGT
**Cr_TC57924**	GTCGAAGAATGACCAAACAAAGAA	GCTAGCGTGCGAGATGCTATT
**Cr_TC33880**	CCAATTTGTCATTCCTGGGATAA	CCCGAACCACTTGACGACAT
**Cr_TC02224**	GAAGCAAACCAGCACCAGAGT	TGATTCAAATACCTGGGCTTCCT
**Cr_TC07184**	ACGAAATTTGGGCAGCAGTT	CCATTTAAAAACATCCCCATTGTT
**Cr_TC51001**	TTCTGTTGCTGTTGCTTTTGGT	TCCGTACAATCTTGGCGAAAC
**Cr_TC07063**	CCAACTCAGGCCATACATTCTCA	AGACGATGACCAGTGCATTGAA
**Cr_TC33950**	GATCTTTCTCCTACTACTCCTCGTCAA	GTTAGCCCAAGAATTCGATTTCA
**Cr_TC57680**	TTGGTACAGCACGCAAAGGAT	ATTTGGATATGGAGGAGAAGTTTGG
**Cr_TC35401 (Actin)**	CCATCTCCAGAGTCCAGAACAAT	CCATTCAAGCTGTTTTATCCCTCTA
