# Supplementary material for: Transcriptome Analysis of Catharanthus roseus for Gene Discovery and Expression Profiling
Source: PLoS One. 2014 Jul 29;9(7):e103583. doi: 10.1371/journal.pone.0103583 (PMC4114786; doi:10.1371/journal.pone.0103583)

Fig. S7. Graphical view showing biological process gene ontology term enrichment in up-regulated genes in (A) leaf and (B) root. The GO enrichment was performed using BiNGO. Node size is proportional to the number of genes in each category and shades represent the scale denoting significance level (white- no significant difference).

(A) Leaf

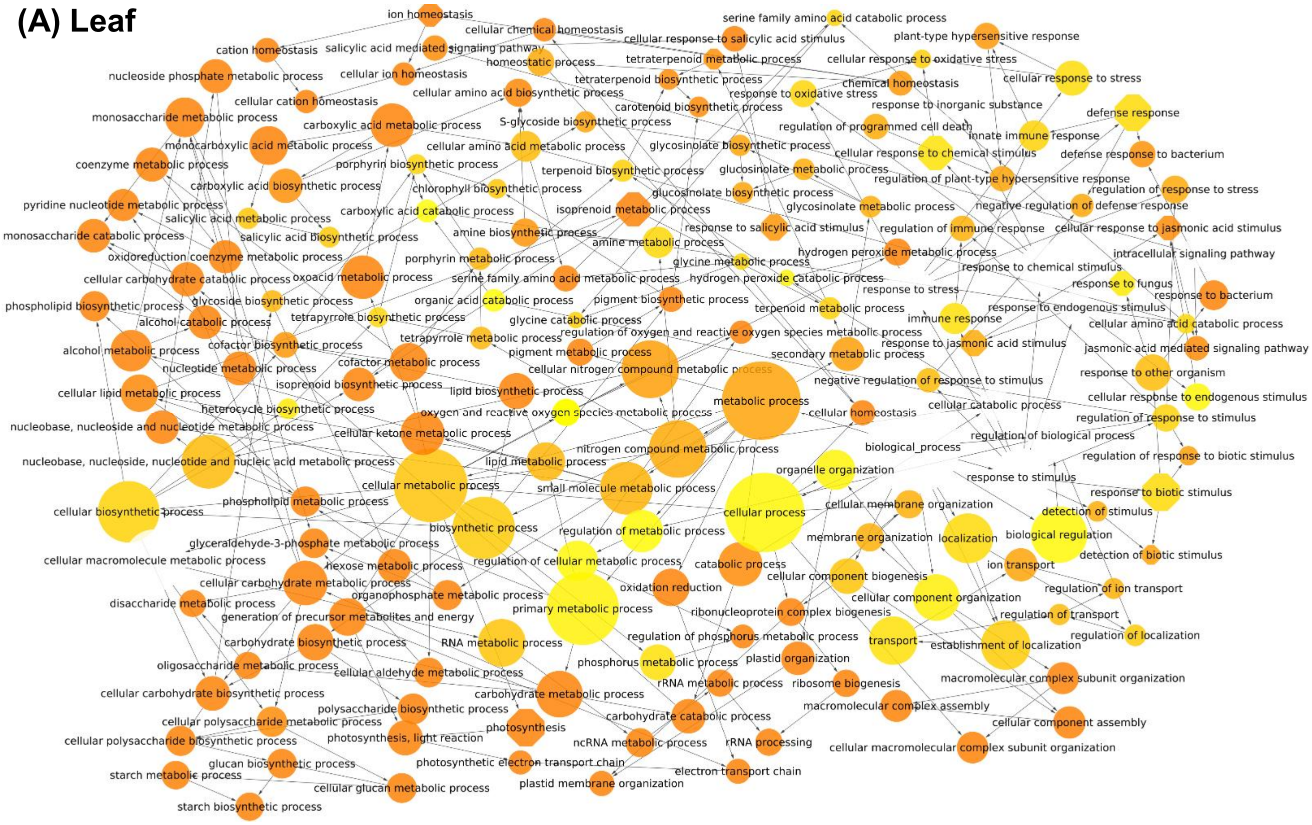

(B) Root

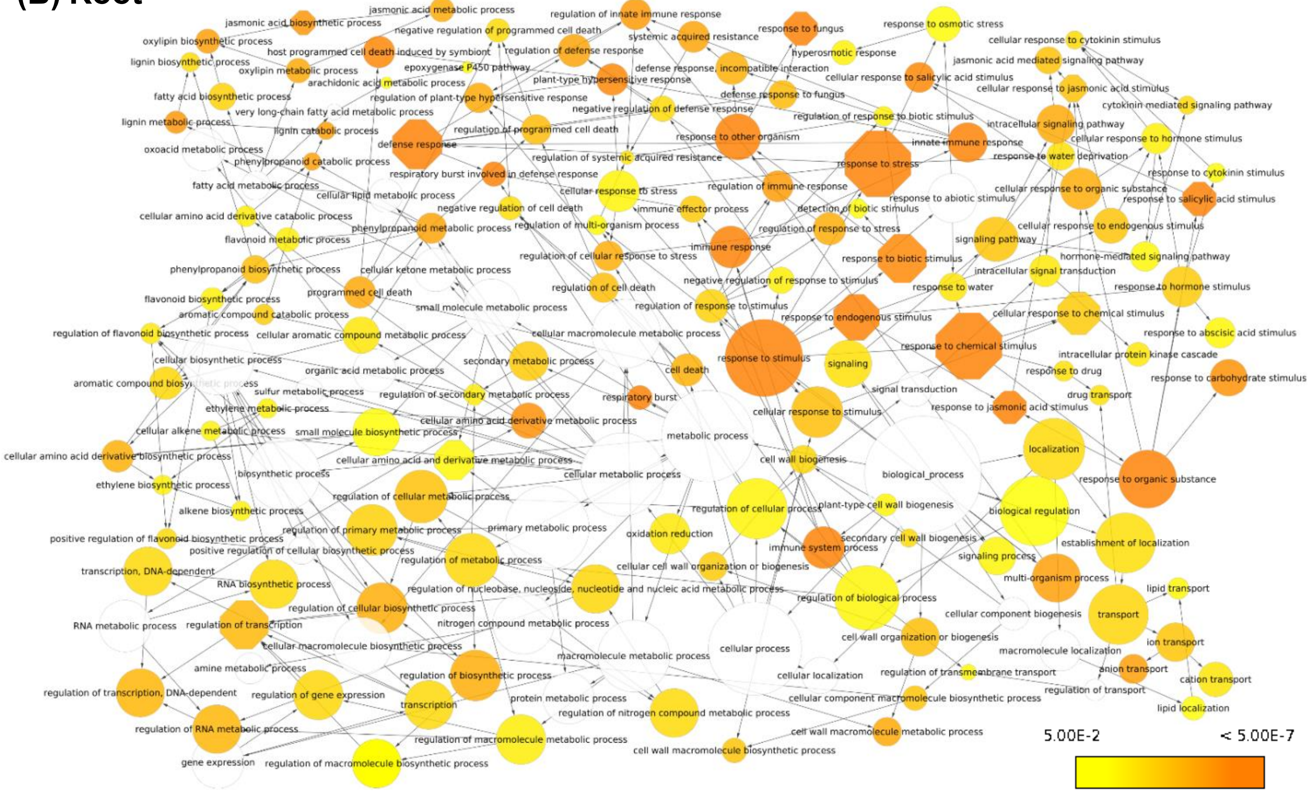

Supplement: Figure S7 — Graphical view showing biological process gene ontology term enrichment in up-regulated genes in (A) leaf and (B) root. The GO enrichment was performed using BiNGO. Node size is proportional to the number of genes in each category and shades represent the scale denoting significance level (white- no significant difference). (PDF) [file pone.0103583.s007.pdf]
